# Interviewing adolescent girls about sexual and reproductive health: a qualitative study exploring how best to ask questions in structured follow-up interviews in a randomized controlled trial in Zambia

**DOI:** 10.1186/s12978-021-01318-1

**Published:** 2022-01-15

**Authors:** Katja Isaksen, Ingvild Sandøy, Joseph Zulu, Andrea Melberg, Sheena Kabombwe, Mweetwa Mudenda, Patrick Musonda, Joar Svanemyr

**Affiliations:** 1 Centre for Intervention Science in Maternal and Child Health (CISMAC), Oslo, Norway; 2 Centre for International Health (CIH), Department of Global Public Health and Primary Care, University of Bergen, Bergen, Norway; 3 School of Public Health, University of Zambia, Lusaka, Zambia; 4 School of Education, University of Zambia, Lusaka, Zambia

**Keywords:** Adolescents, Sexual and reproductive health, Zambia, Survey methods, ACASI

## Abstract

**Background:**

Numerous studies have documented inconsistent reporting of sexual behaviour by adolescents. The validity and reliability of self-reported data on issues considered sensitive, incriminating or embarrassing, is prone to social-desirability bias. Some studies have found that Audio Computer-Assisted Self Interviewing (ACASI) that removes the personal interaction involved in face-to-face interviews, decreases item non-response and increases reporting of sensitive behaviours, but others have found inconsistent or contradictory results. To reduce social desirability bias in the reporting of sensitive behaviours, face-to-face interviews were combined with ACASI in a cluster randomized trial involving adolescents in Zambia.

**Methods:**

To explore adolescent girls’ experiences and opinions of being interviewed about sexual and reproductive health, we combined Focus Group Discussions with girl participants and individual semi-structured interviews with teachers. This study was done after the participants had been interviewed for the 6^th^ time since recruitment. Young, female research assistants who had conducted interviews for the trial were also interviewed for this study.

**Results:**

Respondents explained often feeling shy, embarrassed or uncomfortable when asked questions about sex, pregnancy and abortion face-to-face. Questions on sexual activity elicited feelings of shame, and teachers, research assistants and girls alike noted that direct questions about sexual activities limit what the participant girls may be willing to share. Responding to more indirect questions in relation to the context of a romantic relationship was slightly easier. Efforts by interviewers to signal that they did not judge the participants for their behavior and increased familiarity with the interviewer reduced discomfort over time. Although some appreciated the opportunity to respond to questions on their own, the privacy offered by ACASI also provided an opportunity to give false answers. Answering on tablets could be challenging, but participants were reluctant to ask for assistance for fear of being judged as not conversant with technology.

**Conclusion:**

Strategies to avoid using overly direct language and descriptive words, asking questions within the context of a romantic relationship and a focus on establishing familiarity and trust can reduce reporting bias. For the use of ACASI, considerations must be given to the context and characteristics of the study population.

## Plain language summary

Numerous studies have found that adolescents may not answer truthfully or consistently when questioned about issues such as sexual activity, abortion and pregnancy. Such issues are considered sensitive, incriminating, or embarrassing, and answers may be influenced by a fear of being misjudged or sanctioned. In an attempt to collect more reliable data on sexual behaviour and childbearing, we combined face-to-face interviews with Audio Computer-Assisted Self Interviewing (ACASI) in a research trial involving adolescents in Zambia. In this study we aimed to explore adolescent girls’ experiences and opinions of being interviewed about sexual and reproductive health when participating in the trial. We combined group discussions with girl participants and individual interviews with teachers. Participants said they felt shy, embarrassed or uncomfortable when asked questions about sex, pregnancy and abortion face-to-face. Teachers, girls and interviewers alike noted that very direct questions limit what the participants may be willing to share. Efforts by interviewers to signal that they did not judge the participants for their behaviour, and repeated interviews with the same interviewer reduced discomfort over time. Some liked ACASI whereas some said it made it easier to give false answers, and answering on electronic tablets could be challenging. To reduce embarrassment and increase the likelihood of honest answers we recommend avoiding overly direct language and descriptive words, and to relate questions about sexual activity to a romantic relationship. Interviewers who are able to establish familiarity and trust can make participants more comfortable.

## Introduction

Adolescent sexual and reproductive health is a global public health concern which has received growing interest and increased research attention over the last two decades [1]. To address important issues such as early pregnancy, child marriage, adolescent maternal mortality, HIV and sexually transmitted infections (STIs), it is vital to gather evidence on adolescents’ attitudes and behaviours towards sex and reproductive health. Commonly, data on attitudes and behaviour is collected through self-reporting. However, numerous studies have documented inconsistent reporting of sexual behaviour by adolescents [2–4]. The validity and reliability of self-reported data on issues which are sensitive, incriminating or embarrassing, is prone to social-desirability bias and recall bias [5, 6]. This is the case particularly in contexts where adolescent and/or pre-marital sex is socially disapproved or even taboo and may be sanctioned by parents, teachers and the community because young people place great emphasis on peer acceptance and fitting in [7]. Some studies have documented, for example, that many young people who tested positive for HIV or sexually transmitted infections (STIs) reported never having sex, or that students who reported sexual activity later claimed to be virgins [8, 9].

The Audio Computer-Assisted Self Interviewing (ACASI) technique has gained some popularity amongst researchers due to the privacy it affords the respondent. When using ACASI, respondents receive and respond to questions using a handheld computer or tablet, which removes the personal interaction involved in face-to-face interviews, and this has been found to reduce socially desirable responses. Encouraging evidence for increased reporting of sexual risk behaviour using ACASI was found in USA in the 90 s and 2000s [10–12], and studies in sub Saharan Africa suggest that ACASI, in comparison to self-administered questionnaires on paper, increases reporting of sexual activity [3, 6, 13]. A review from 2010 of studies conducted in low and middle income countries concluded that there was strong evidence that computer-assisted interviews decreases item non-response and increases reporting sensitive behaviours [14]. However, other studies on the ACASI technique have found inconsistent and/or contradictory results in terms of differences in reporting between face-to-face interviews and ACASI [15, 16].

In the Research Initiative to Support the Empowerment of Girls (RISE) we combined face-to-face interviews with ACASI to reduce social desirability bias in the reporting of sensitive behaviours. RISE was a cluster randomized controlled trial that aimed to measure the effect of economic support for adolescent girls, sexuality education and community dialogue on childbearing rates amongst adolescent girls in Zambia [17]. Throughout the duration of the trial, the participants were interviewed twice per year. When interviewers reported that they experienced that many of the participants appeared to be nervous during the interview and sensed that they were not being completely truthful when responding to sensitive questions, we combined the face-to-face interviews with an audio computer assisted self-interview (ACASI), allowing the participants to respond to some questions on their own using a tablet and headphones from the fourth follow-up interview. Preliminary analyses of the data from the fourth follow-up interview on sexual activity, contraceptive use, and pregnancy from the face-to-face and ACASI portions revealed some inconsistencies. We expected to find that some of those who denied being pregnant or having sex in the face-to-face interviews would admit having been pregnant or sexually active in the ACASI since the latter gave them full privacy. However, we were surprised to find that some who denied being pregnant or having sex in the ACASI, admitted it in the face-to-face portion. The objective of this study was to explore adolescent girls’ experiences and opinions of being interviewed about their sexual and reproductive health (SRH) and their recommendations for how interviews should be done to make young girls open up about their sexual experiences, marriage and childbearing.

## Methodology

### Background information: the RISE trial

The participants in the RISE trial were girls who were enrolled in grade 7 in 2016, in 157 rural basic schools across 12 districts in Southern and Central provinces of Zambia. The age range of the participants at enrolment was 10–25 years old, with the mean age being 14.1 years old. Schools were randomly allocated to one of three groups: (1) *Control Group*, (2) *Economic support group* with cash transfers to girls and their families and payment of school fees or (3) *Combined intervention group* receiving the same economic support combined with sexuality education and community meetings targeting norms related to education and SRH amongst parents and community members [17].

The RISE interviews were carried out by young, female research assistants (RAs) between the ages of 17 and 25. They were recruited locally, and trained in appropriate interview techniques, including confidentiality, sensitivity and how to make respondents feel at ease and comfortable. Given the age group (adolescents) and sensitivity of the topics being covered, special emphasis was placed on displaying empathy and neutral attitudes to the participants. All the trainings included sessions where the RAs practised the interviews and received individual feedback on their interviewing skills. The RAs were also given contracts that emphasised the importance of professional and respectful conduct and confidentiality. The RAs were equipped with hand-held tablets which contained the interview questions and response options. Furthermore, scripts for introductory or prompting purposes were included in the questionnaires to ensure that the interview was as standardized as possible. All questionnaires were translated to local languages and back translated for verification of accuracy and pretested. Sensitive questions came at the end of the interviews with the hope that by then they would feel more comfortable with the situation.

The girls recruited for this study had completed their fourth follow-up interview when the RA asked them whether they were willing to participate in a focus group. There were no refusals. The discussions were conducted in empty classrooms and interviews with teachers in the teacher’s office, ensuring that others could not hear what was being said. None of the interviewers were at any stage involved in the delivery of the RISE intervention. The baseline and first three follow-up interviews were interviewer-administered (face–to-face or via phone for those who had moved). From the fourth follow-up round an ACASI section was added to the biannual interview for those interviewed face-to-face. Before starting the ACASI portion of the interview, each participant was given a short orientation on how to respond to the ACASI questionnaire on the tablet. The RA gave the participant an opportunity to practice before moving away to give the participant privacy. The RAs were instructed to move a few meters away and wait in front of the participant to prevent them from seeing the responses but still be able to detect whether they appeared to have technical challenges responding to the ACASI so they could offer advice.

### Procedure

The data for the current study was collected in November and December 2018. Two researchers – one female lead researcher from Norway and one supporting female researcher from Zambia accompanied the RAs to the schools where they were conducting follow-up interviews. A total of seven schools from the RISE project were included in the study: three from the control group, two from the economic support group and two from the combined intervention group. The selection of schools was a convenience issue since they were schools due to have follow up interviews. Eight focus group discussions (FGDs) (two in one of the sites) were held with RISE participants with an average of six participants in each group. FGDs with RISE participants were held in classrooms at the school after they had completed their biannual follow-up interview. Discussions lasted between 45 and 60 min and were conducted in local language by the supporting researcher using a semi-structured discussion guide. RISE participants were asked how they would describe any aspects of the interview they found uncomfortable or strange to a friend. This was done to provide the participants with an *external* base for their answers which allowed them to avoid speaking *directly* about their *own* experiences and feelings [18]. The participants were also asked what they thought could make some participants respond inconsistently or untruthfully to questions about sexual activity, pregnancy, and marriage. To facilitate the discussion, the groups were presented with a vignette about a fictional RISE participant, Mutinta, who was described as ‘pregnant but claims never to have had sex’, and participants were asked to explain how she may respond to a particular question and why. At the end of the discussion, participants were asked how interviews could be improved to ensure more honest responses, including how to ask particularly sensitive questions, whether they thought face-to-face interviews or ACASI was better, their preferred interview location, and who would be the best person to conduct face-to-face interviews. The FGDs were audio recorded in order to capture properly what all the participants said.

Individual interviews were held with five RAs and six teachers who were the designated RISE study focal point at the school. The RAs and RISE focal point teachers were deemed as important informants due to their close involvement in the project. The five RAs were informally interviewed about difficulties they faced, how they dealt with these, and whether they believed the respondents were responding truthfully, and if not, why not. They were also asked if they had any specific suggestions for how interviews could be improved. The interviews with RAs were recorded as notes since they were done in the car with background noise on the way to the field, and to make the RAs as relaxed and open as possible. We wanted to avoid that they had any feelings of being criticized or that the information could be used against them.

The lead researcher interviewed six RISE focal point teachers (of whom three were also guidance and counselling teachers) and one head teacher. The RISE teacher at one of the schools was not available. The interviews were conducted in English using a semi-structured interview guide, lasted on average 30 min, and were recorded on a digital voice recorder. The teachers were asked whether they had heard any complaints or other feedback about RISE interviews from the participants, and of their opinion about the formulation of questions regarding SRH and how they themselves would have asked such questions to pupils.

The number of data collection points was determined by the number of schools that were being visited by the RAs during the 2-week period that the researchers were travelling with them. When interviews were analysed, we also found that we had reached saturation.

### Analysis procedure

The FGDs were transcribed and translated in verbatim directly to English by the supporting researcher, and the teacher interviews conducted in English were transcribed by the lead researcher. The recordings for two FGDs and one teacher interview were lost due to problems with the recording device, but detailed notes were taken for these immediately after they were completed. At the end of each data collection day, the two researchers discussed the FGDs and interviews that had been conducted that day. This allowed for the identification of emerging themes that could be explored further and for adjustments of the interview and discussion guides. The transcripts and field notes were entered in NVIVO 12.0 for coding by the lead researcher.

The thematic analysis was guided by the objectives of the study which were to gain insight into the reasons for inconsistent interview responses and to identify ways to increase the validity of the interview data. We applied a mix of deductive coding based on pre-established themes and inductive coding based on themes emerging from the data. The first stage of analysis concentrated on identifying the aspects of the interview procedure which may cause a RISE participant to respond inconsistently or contradictory. In the second stage of analysis, our focus moved to the suggestions and insights given by FGD participants, interviewers, and teachers on how to make the interview process more conducive. The final analysis stage focused on the experiences and opinions of FGD participants and interviewers on using the ACASI technique in the RISE study context.

### Ethics

All the RISE participants had provided consent to be part of the RISE project wherein this substudy falls. Likewise, the RAs were part of the RISE project and gave verbal consent to be interviewed about their work. All teachers were briefed in detail about the purpose of the study and informed consent was given verbally. Ethical clearance was obtained from the University of Zambia Biomedical Research Ethics Committee (ref no 021- 06-15) and the Regional Ethics Committee of Western Norway (ref no 2015/895) before the start of the trial in 2015. All the data for this qualitative study—transcripts, field notes and digital voice recordings—were uploaded and stored on a secure server (owned by the University of Bergen). No names or other information that would allow identification of the participants were recorded.

## Results

The findings are categorized in line with the three predefined themes: Barriers to truthful reporting of sexual behaviour by adolescent girls; suggestions to address these barriers; and observations on the ACASI method.

### Barriers

#### Questions about sensitive topics

The respondents often explained feeling shy, embarrassed or uncomfortable when asked questions about sex, pregnancy and abortion face-to-face. The interviewing RAs confirmed that many of the girls appeared *visibly* uncomfortable when they were asked these questions. They tended to look down, hunch their shoulders and remain silent. Responses to detailed questions about the frequency of sexual activity, sexual partners, pregnancies and/or contraceptive use frequently gave RAs a feeling that the full truth was withheld. FGD participants confirmed the questions caused discomfort, regardless of whether they admitted having engaged in sexual activity or not*: “Even that question, ‘have you ever had sex’ is embarrassing especially if you have done it before, it just puts you off” (Girl, FGD).* The participants expressed that they suspected that girls who had engaged in sexual behaviour, were particularly likely to experience feelings of shame.

Questions about sexual behaviour were perceived to be the most difficult to answer. Girls in a group elaborated a bit on this:

Girl**:** *Have you ever had sex? Have you ever given birth? Do you have a child? Are embarrassing [questions] but the most embarrassing is the sex question, so it is difficult to answer.*

Researcher: *So why do you think this particular question is embarrassing? Because you are saying that asking if you have a boyfriend is not difficult to answer.*

Girl**:** *Because they will start thinking of me as a girl who opens her legs (girls laugh) Ah!* (FGD 2)

Another group gave several reasons why girls would lie about ever having had sex: “[the girl’s]self-esteem is low”; “Some girls get offended if you ask them that how many men have you ever slept with”; “some fear that their parents may find out at home and tell the Rise teacher at school”; “fear of being laughed at”, “fear she may be reported to people”; “not just understanding the questions”, “maybe they think that when you meet them [RAs] in the streets, you will be looking at them with an eye that you know about them” (FGD 1).

Questions around pregnancy also made the respondents uncomfortable:

Researcher*: So, are there any questions that make you feel uncomfortable? Maybe even embarrassed?*

Girl 1*: (laughs) Those ones whereby she is asking you if you have ever been pregnant, have you ever given birth? They are embarrassing but more embarrassing if you are actually pregnant.*

*Researcher 2: Can I ask why they are embarrassing?*

Girl 2*: If you have been pregnant it is embarrassing or shameful.* (FGD 2)

Questions on sexual activity elicited feelings of shame and teachers, RAs and girls alike noted that they limit what the participants may be willing to share: *“Some people who respond to these questions do not tell the truth. They lie mainly because they feel guilty and ashamed and don’t want to be discovered as a prostitute”* (Girl, FGD). The girls reported that they are continuously reminded and ‘warned’ by parents, teachers and the community that it is wrong and dangerous to have sex before marriage. This may result in an internalization of shame, guilt and negative feelings, which may have direct implications for the way they respond to sex-related questions.

Teacher*: because these girls they know that when they are doing these things* [sexual behaviours]*, they know it’s not right and they are advised not to be doing these things. So when they say that they do it, they feel guilty because they are doing the wrong thing or have been told not to be doing sex before marriage so that they don’t contract STIs and HIV and they don’t become pregnant. So, when they are asked to say ‘Have you ever had sex?’, they are going to deny because they will know now they are not trusted.* (Interview with RISE teacher)

Another teacher suggested that questions about having a child are less likely to create feelings of shame than questions about sexual behaviour or pregnancy.

Researcher*: you said with sex there was guilt and shame, is that the same with having a child? Is there shame?*

Teacher*: sometimes, for having a child they are not shy, they are going to tell you straight away, that one is not very difficult. The most difficult is where you ask them, do you have a boyfriend, have you ever had sex? Are you pregnant? They hide! But if you ask them if they have a child, they don’t usually hide that.*

When FGD participants were asked why some respondents would report having a child in the face-to-face interview but respond ‘no’ to the questions referring to sex in ACASI, they explained that it is easier to say that they have a child because, “it’s like a lighter version” of saying sex. The shame, guilt and perceptions of promiscuity associated with ‘sex’ amongst these adolescent girls, made them want to distance themselves from it.

Teachers and RAs would also prefer to avoid the word ‘sex’ since the word itself is source of embarrassment. It is rarely, if ever, used in the local public sexual and reproductive health discourse. Although the act of sex is implicit when talking about having a child or pregnancy, they considered that using the word itself creates a risk that participants alter their response.

Researcher*: we have some situations where a girl will say she has a child and then if you ask her later if she’s ever had sex, she’ll say no*

Teacher*: yes, she’ll say no. (laughs). Why? Because of sex! Aha!! Because of sex…. Just that word ‘sex’, it means ‘a man in me’, just that! She will feel shy, unless another term is used.* (Interview with RISE teacher)

Questions about miscarriages and stillbirth were perceived as offensive and upsetting despite of RAs being trained to approach such sensitive topics with care and empathy. It appeared that this was partly worsened by the structure of the interview which meant that a number of these questions were repeated (they were asked face-to-face as well as in the ACASI portions).

Girl*: But others are not fine with these questions. Some may end up slapping you over these questions* [about miscarriage/stillbirths]*, it may be that she was once pregnant but had a stillborn, then you are asking her these questions repeatedly. They may not be in the mood or they may respond to you in a way that is not good just to offend you.* (FGD 2)

Reactions to questions relating to induced abortion were particularly strong as there is a lot of shame associated with abortion.

Researcher*: Why do you think these questions [about abortion] are difficult to answer?*

Girl: *Because they feel like if they say the truth, someone is going to tell someone and so on. Abortion is not acceptable in society. It’s actually embarrassing to be known as a person who has aborted.* (FGD 4)

#### Feeling judged

Fears around confidentiality and anonymity of answers were frequently cited by RAs and teachers as a reason for participants not responding truthfully to sensitive and/or taboo questions. In contrast, RISE participants explained that the questions about sex and abortion made them feel judged by the interviewer. A girl who has a child and is asked whether she has ever had sex, may answer ‘no’ because she is afraid of being laughed at, according to participants in one of the groups (FGD 3). Questions about unacceptable behaviours made respondents believe they were suspected of engaging in that sort of behaviour, implying that the interviewer had made a negative evaluation of their character.

Girl 2: *There is just one question that I was asked, and I thought to myself ‘what has made this person to ask me this question?’*

Researcher*: Which question is that?*

Girl 2*: The questions ‘Have you ever aborted?’. I was disappointed because it is not allowed to abort and I thought the person asking me was looking at me as if we do not respect ourselves.* (FGD 5)

### Approaches to overcome barriers

The following section describes the various respondents’ suggestions for overcoming barriers in collecting self-reported SRH data. Four key strategies were mentioned by all three respondent groups.

#### Establish familiarity and comfort

As outlined above, the sensitive nature of several of the questions meant that respondents required a great deal of confidence in the interviewer if she was to answer honestly. The girls claimed that they felt comfortable being interviewed by the trial RAs and emphasised the importance of ‘getting to know’ and developing a close relationship with them. This familiarity was created in the informal conversations prior to commencing the interview, throughout the course of each interview, and through repeated interviews (conducted every 6 months) by the same person. The respondents became gradually more comfortable with the interview process as well as the RA.

Girl 1*: At first I was uncomfortable and shy because I was not used to being asked these questions by a stranger, a person you don’t know completely, asking you these questions*

Researcher*: What made you not be shy afterwards?*

Girl 1*: Because I became used to being asked such questions. You can only talk about these things to people you are close to.* (FGD 3)

We were curious whether changing RAs between interview rounds would increase the feeling of anonymity amongst the RISE participants, but this did not appear to be the case. As can be seen in the quote below, when the RAs returned for subsequent interviews, the girls felt they had already heard their secrets.

Girl*: The thing is that we would like to be interviewed by the same person because we are now used to her as opposed to someone we haven’t met. We also feel like she cannot release our information to people and we are not scared of her.* (FGD 6)

RAs often used their repeated visits to reinforce the confidentiality of the interviews. Several of them said things like: *“We’ve been coming here for a long time—have you ever heard of anyone being ‘told on’ or information being shared?”.* Familiarity with the respondent also made it easier to judge the reliability of her answers and the appropriate approach to assume. For example, if the participant was pregnant in the previous interview, the RA could be more attentive when asking questions around childbirth or miscarriage.

Teachers and FGD participants suggested that the school was considered the ‘safest’ location to conduct the interviews. The presence of community members at the clinic could create a risk of rumours and curiosity, according to the girls. At their homes, the presence of parents seemed to prevent speaking about sex and reproductive issues. Several of the RAs had experienced that the girls avoided being found in their homes when they arrived.

When RAs were able to normalize sex and pregnancy by giving examples from their own lives, it seemed to be an effective way of making a respondent comfortable enough to answer questions on this topic.

Girl*: Another way to make us comfortable so that we can answer truthfully, is to tell the person who interviews us to open up freely. They can say that ‘Even I have slept with a man and know everything that happens between a man and a woman. So whatever girls go through, I have also gone through that, so feel comfortable.’* (FGD 1)

RAs occasionally referred, without negative connotations, to a young family member who was pregnant, which was meant to signal to the respondent that they had no negative judgement towards that behaviour.

#### Contextualise the question

When reviewing the questionnaire items with respondents, they noted that the sequencing and formulation of the questions may impact on the respondent’s reaction to the interview. The comments of the respondents suggested that in the follow up interviews, sex-related questions were not appropriately contextualised in the frame of a stable and/or romantic relationship. All three respondent groups emphasized the need to present questions around sexual activity with the assumption that it was with a boyfriend.

Researcher*: Ok, if you want to ask a friend if she had sex, how would you ask her?*

Girl*: I can ask her, ‘Do you have a boyfriend. Or are you dating?’ Then that’s when you ask her if she had slept with him.* (FGD 6)

*“In fact they should be asking these questions like do you have a boyfriend and do you have a child together, so as to avoid the awkwardness or just removing the sex question*.” (FGD 2)

A head teacher also suggested that the interviewed girl should be eased into the question.

Researcher: Is there some way to make it less direct?

Head Teacher: Sometimes you start asking ‘Do you have a boyfriend?’ And from there ‘From the time that you met that boyfriend, have you done anything with him?’ Or maybe ‘What type of stories do you share with your boyfriend, and at what time do you meet your boyfriend?’ Such things. Maybe ‘Do you see your boyfriend in the presence of your mother? Or maybe you have got some places where you meet this boyfriend?’ Just like those minor questions before you hit to the real point of ‘Have you ever slept with your boyfriend?’. (Interview with head teacher)

### Observations on the ACASI method

When the girls shared their opinions about the ACASI interview, the privacy that it provided was often highlighted as a positive feature. However, this privacy also provided an opportunity to give false answers:

Girl 1: *Ok, the tablet is fine to those who are shy to talk face to face with the interviewer, even though there is a chance they can lie. For instance, if she is pregnant when answering on the tablet, she can say ‘no’. But when asked during face to face, she admits it because you have seen for yourself that she is pregnant.* (FGD 3)

The lack of support and contact with the interviewer was a clear drawback of ACASI.

Researcher*: Which one do you prefer? The tablet or face to face?*

Girl 2: *I prefer being asked live [face to face]*

Girl 3*: We prefer face to face because we are interacting with a person who can read our expressions and use better judgement. And also if you did not really understand the questions, you can ask if you are not clear.* (FGD 3)

RAs and FGD participants noted that the language and dialect of the audio recordings in ACASI could be challenging, but participants hesitated to ask for assistance for fear of being judged as not conversant with technology: *“Sometimes we feel shy because we feel like they will tell someone that we don’t know how to use the tablet”* (Girl, FGD 3)*.* Indeed, many of the informants—both the girls and RAs—suggested more support and training should be given to those RISE participants who struggled with the ACASI technique.

When the FGD participants were asked why some girls may prefer the face-to-face interview, they suggested that with the ACASI, being presented with a written text, a voice recording and being asked to select a response at the same time, could be confusing.

Girl 1*: Ooho! It’s reading sometimes which is the problem. Sometimes you are reading and at the same time you are listening from the headphones, and then you have to press at the same time. You end up making a mistake pressing ‘Yes’ to the question when you meant ‘No’.*

Girl 2*: But the tablet asks which language you understand. It’s just that others do not know or hear the instructions where to press. So, others find it difficult to use.*

Researcher*: So sometimes you put an answer you didn’t want to put?*

Girl 2*: Yes.*

Researcher*: So, what can we do to make it easier on the tablet?*

Girl 1*: Maybe they should come and teach those who find it difficult for some time, not just a short time.* (FGD 1)

Several RAs felt that some girls had rushed through the questions in the ACASI portion and gave examples of respondents who completed the ACASI portion of the interview in less than five minutes when on average it took 10 min to complete. FGD participants suspected that some girls may have rushed through the ACASI portion because they were uncomfortable with the method and/or technology. All respondents (RAs, FDG participants and teachers) emphasised the importance of offering continuous clarification and support throughout the interview even when not requested by the participant herself.

## Discussion

This study found that questions about sex, pregnancy and abortion made the female adolescent trial participants uncomfortable and elicited feelings of shame. These reactions were common despite efforts to mitigate them by the use of female research assistants of similar age who were instructed and trained to normalize the questions and topics. Repeated questions about private, sensitive issues such as stillbirths could even elicit feelings of anger. The reasons given for underreporting sexual activity in this study were largely in line with findings from previous studies; namely shyness, embarrassment, social desirability, concerns around confidentiality and fear of penalties and social consequences [8, 9, 19]. However, participants reported that their discomfort was reduced over time as they got used to the questions and the same interviewers coming back repeatedly, which allowed them to establish feelings of familiarity and trust. A strategy used by some RAs to help build this trust and reduce the embarrassment and shame was to normalize behaviour such as premarital sex, which made the participants understand that the interviewer did not consider their behaviour unacceptable.

Throughout the FGDs and interviews it became apparent that sex-related questions which had been used in the follow-up interviews were perceived to lack any reference or association with love, trust, or intimate and stable relationships. Not surprisingly therefore, all the informants advised the researchers to introduce sexual activity questions in a context which assumed that they were performed with a boyfriend. Similar observations on the need for such contextualising have also been made by interviewed sex-workers in Malawi and South Africa who described their relationship with clients in terms which connoted premarital love and trust; not simply transactional relations [20, 21].

Some participants highlighted that the use of the word ‘sex’ can affect how girls answer certain questions. This may be explained through other studies from Zambia which found concerns that the local terms related to sex emerged as more insulting than the English ones (Zulu et al. 2019). This suggests that other ways of asking sex-related questions should be considered such as asking about ‘boyfriend’ instead of ‘sex partner’, ‘sleeping with’ instead of ‘having sex’, or ‘dating’ instead of ‘sexual relations’. Although the use of local euphemisms and culturally appropriate terminology may require additional preparation and adjustment of research tools, it can make the interview less awkward for both the researcher and the respondent. Alternative expressions such as ‘slept with’, not only distances the respondent from the act of sex but as found by Langhaug and colleagues (2010), it also avoids the embarrassment of hearing sexual terms being read out loud by the interviewer. Formatting of questions in a vague manner, however, contrast with typical survey design guidelines that recommend clear and unambiguous questions to avoid misunderstandings and to increase the validity of responses [22, 23].

The RISE participants explained that the questions about sex and abortion made them feel that they were judged by the interviewer and suspected of engaging in socially unacceptable behaviour. They seemed most concerned about being associated with promiscuity and/or prostitution. This was also seen in a study of reporting of sexual behaviours amongst South African women, which found that 32% of respondents purposefully under- or overreported certain sensitive behaviors to avoid criticism or to seek praise [19]. As pointed out by Poulin, the literature indicates “that when young people misreport on their sexual behaviour, they do not do so as much from “response error” as from active attempts to manage their identities” [24]. A similar study of Zimbabwean adolescents found that young women struggled to admit to having sex if it was phrased in a manner which suggested that they had initiated it [25]. These concerns seem to be grounded in a cultural script suggesting that women should be passive recipients of sex [26], and sexual activity should happen within the context of a relationship [25, 27].

The participating girls stated that the school was the most preferred place for interviews. Having interviewers coming home to them created a risk of embarrassing situations with family members asking why they were talking to strangers about sexuality related issues. They also feared that being seen at the health clinic could cause negative rumours. These findings should be seen in relation to other studies in the region who have reported that adolescents rarely communicate with their parents about sexuality and most often seek to hide any sexual relations or interest in boys. Studies have also suggested that adolescents may avoid health clinics out of fear that fellow community members will suspect them of seeking contraception [28–30].

Discussions around the audio computer assisted (ACASI) section of the interview seemed to confirm previous claims around the increased privacy and anonymity afforded by the technique. The use of ACASI was welcomed by some, but the participants did not believe that adolescent girls would necessarily be more truthful when left to respond to sensitive questions on their own. Some studies in low-income contexts have shown higher rates of self-reported sexual activity using ACASI [14, 31]. For example, a study of high-school students in Malawi found that ACASI led to more reporting of highly stigmatised behaviours related to sex, thereby revealing a more complex picture of adolescent sexuality in comparison to face-to-face interviews [6]. Other studies have not found the same benefit of the method [32, 33]. Participants in this study explained that factors such as language issues and unfamiliarity and discomfort with the use of tablets may lie behind some of the inconsistencies found with ACASI in the data from the fourth follow-up round, and that trial participants may have avoided asking for help because they were afraid of being perceived as ‘slow’ by the RAs. Indeed, studies suggest that the fear of such judgements is common. In an ACASI study with Zimbabwean adolescents, participants admitted that they sometimes responded even if they did not understand the question, because it was embarrassing to ask for clarification [13].These observations align with previous research in Malawi and Kenya which found that participants in rural areas were suspicious, apprehensive and at times hostile towards the highly computerized ACASI method [6, 34]. As Mensch and colleagues point out, face-to-face interviews may be more appropriate in certain contexts because interviewers can take the time to ensure, encourage and assist the respondent when required [6]. Demographic and social characteristics can also influence whether ACASI is a suitable method for self-reporting. For example, Zimbabwean women with higher levels of education reported significantly less problems using ACASI than those with only primary school level; 10% versus 53% respectively [31]. Similarly, studies that advocate strongly for the suitability of ACASI in low-income contexts are typically based on findings from older adolescent samples (18 years and older) [14, 31]. Furthermore, a study amongst 15–19 year old Indian respondents found that females reported less sexual activity in ACASI than face-to-face, whereas males reported more in ACASI [33]. Similar gender-specific response patterns have been found amongst adolescents in Tanzania [2] and may suggest that adolescent girls feel more comfortable in reporting SRH when they have more interaction with the researcher than boys do.

Research on adolescent SRH may need more careful consideration of the value, relevance and wording of detailed questions about sexual behaviour. If a study *does* require in-depth details about adolescents’ sexual activities, careful piloting of alternative wording and combinations of data collection methods may increase the validity of the findings. Comparative studies suggest that culturally specific interactive interviews [33] and in-depth interviews [35] yield higher reporting of sexual activities such as homosexual acts, multiple and/or non-regular partners and commercial sex than face to face interviews with standardized questionnaires. Replacing self-report questionnaires with such methodologies may have implications for the scope, sample size and measurability of the data, however, it may yield benefits such as increased depth and reliability of the findings.

### Strengths and limitations

A strength of this study is that we triangulated the responses from participants, teachers, and the RAs themselves. Their observations and suggestions were similar, thus giving credibility to the findings. Another strength was the use of two interviewers. One was a white, 37-year-old woman from Norway and the other interviewer was a Zambian 25-year-old female. The latter spoke the local languages the girls were comfortable with and her age was close to the participants. As for the former, her Scandinavian background with liberal and open views about SRHR issues may have enabled her to ask more direct questions than what a local person would feel comfortable doing, and it is possible that the presence of an outsider made it possible to talk about things that are normally regarded as too private. However, direct ways of questioning may be intimidating for participants who are less accustomed to talking about SRH. The identification of the researchers with the main study may have caused concern that any negative opinions about the RISE project would have implications for the support that it provided to the girl or the school, but explicit efforts were made to ensure the participants that they could be honest and that their responses would have no bearing on them or their participation in the RISE project.

Two FGDs and interviews with RAs were not recorded, which may have resulted in some loss of detail or nuance although detailed notes were made as soon as this was discovered. In addition, the transcriber was not fully conversant with the language used in one of the FGDs and quotes may not have been completely accurate.

Since we reached saturation, we believe the findings are transferable to the study population of the RISE project. Due to the special context of the study, the generalizability is uncertain but it is likely that the findings are relevant to other studies on sexual and reproductive health among adolescent girls in sub Saharan Africa.

## Conclusion

This study found that the female adolescent trial participants felt uncomfortable and embarrassed when asked questions about socially disapproved behaviour such as premarital sexual activity and pregnancy, despite repeated experiences of being asked the same questions. Strategies to avoid using overly direct language and descriptive words, asking questions within the context of a romantic relationship and a focus on establishing familiarity and trust can reduce reporting bias. Considering discrepancies in the research on ACASI, it is premature to make generalised claims about its suitability for research in low-income contexts. The findings of this and previous studies suggest that considerations must be given to the context and characteristics of the study population, as well as the time and resources available to familiarize the respondents with ACASI technology.

## Data Availability

The data is available upon request.

## Abbreviations

ACASI: Audio computer assisted self-interview
FGD: Focus group discussion
HIV: Human immunodeficiency virus
RA: Research assistant
RISE: Research Initiative to Support the Empowerment of Girls
SRH: Sexual and reproductive health
STI: Sexually transmitted infection
